# Supplementary material for: Unveiling the Bioactive Potential of the Invasive Jellyfish Phyllorhiza punctata Through Integrative Transcriptomic and Proteomic Analyses
Source: Biomolecules. 2025 Aug 4;15(8):1121. doi: 10.3390/biom15081121 (PMC12383608; doi:10.3390/biom15081121)
Supplement: Supplementary file 1 [file biomolecules-15-01121-s001.zip › Suplementary Material/FileS3/PEPFOLD4/XP_065059637_11091/clusters.html]

 PEP-FOLD clusters


Download archive of all models  here   

|  |  |  |
| --- | --- | --- |
| # | file | sOPEP |
| # cluster 1 - Size: 10 |  |  |
|  | XP\_065059637\_11091-model1 | -59.1752 |
|  | XP\_065059637\_11091-model1.1 | -55.7702 |
|  | XP\_065059637\_11091-model1.2 | -52.7856 |
|  | XP\_065059637\_11091-model1.3 | -49.3335 |
|  | XP\_065059637\_11091-model1.4 | -47.4622 |
|  | XP\_065059637\_11091-model1.5 | -46.9147 |
|  | XP\_065059637\_11091-model1.6 | -46.3905 |
|  | XP\_065059637\_11091-model1.7 | -45.9613 |
|  | XP\_065059637\_11091-model1.8 | -45.478 |
|  | XP\_065059637\_11091-model1.9 | -45.3629 |
| # cluster 2 - Size: 4 |  |  |
|  | XP\_065059637\_11091-model2 | -58.4285 |
|  | XP\_065059637\_11091-model2.1 | -56.349 |
|  | XP\_065059637\_11091-model2.2 | -45.6955 |
|  | XP\_065059637\_11091-model2.3 | -41.3782 |
| # cluster 3 - Size: 1 |  |  |
|  | XP\_065059637\_11091-model3 | -53.2684 |
| # cluster 4 - Size: 1 |  |  |
|  | XP\_065059637\_11091-model4 | -52.6699 |
| # cluster 5 - Size: 1 |  |  |
|  | XP\_065059637\_11091-model5 | -51.8295 |
| # cluster 6 - Size: 1 |  |  |
|  | XP\_065059637\_11091-model6 | -51.525 |
| # cluster 7 - Size: 7 |  |  |
|  | XP\_065059637\_11091-model7 | -48.2299 |
|  | XP\_065059637\_11091-model7.1 | -45.0764 |
|  | XP\_065059637\_11091-model7.2 | -42.867 |
|  | XP\_065059637\_11091-model7.3 | -42.7142 |
|  | XP\_065059637\_11091-model7.4 | -42.3621 |
|  | XP\_065059637\_11091-model7.5 | -41.5585 |
|  | XP\_065059637\_11091-model7.6 | -41.2783 |
| # cluster 8 - Size: 16 |  |  |
|  | XP\_065059637\_11091-model8 | -47.4587 |
|  | XP\_065059637\_11091-model8.1 | -46.6589 |
|  | XP\_065059637\_11091-model8.2 | -46.1351 |
|  | XP\_065059637\_11091-model8.3 | -46.0827 |
|  | XP\_065059637\_11091-model8.4 | -45.976 |
|  | XP\_065059637\_11091-model8.5 | -45.723 |
|  | XP\_065059637\_11091-model8.6 | -44.8433 |
|  | XP\_065059637\_11091-model8.7 | -44.822 |
|  | XP\_065059637\_11091-model8.8 | -44.6918 |
|  | XP\_065059637\_11091-model8.9 | -44.2387 |
|  | XP\_065059637\_11091-model8.10 | -43.704 |
|  | XP\_065059637\_11091-model8.11 | -43.4618 |
|  | XP\_065059637\_11091-model8.12 | -42.245 |
|  | XP\_065059637\_11091-model8.13 | -41.8231 |
|  | XP\_065059637\_11091-model8.14 | -41.775 |
|  | XP\_065059637\_11091-model8.15 | -39.3937 |
| # cluster 9 - Size: 10 |  |  |
|  | XP\_065059637\_11091-model9 | -46.8475 |
|  | XP\_065059637\_11091-model9.1 | -45.6392 |
|  | XP\_065059637\_11091-model9.2 | -43.3783 |
|  | XP\_065059637\_11091-model9.3 | -42.998 |
|  | XP\_065059637\_11091-model9.4 | -42.7584 |
|  | XP\_065059637\_11091-model9.5 | -42.75 |
|  | XP\_065059637\_11091-model9.6 | -42.5856 |
|  | XP\_065059637\_11091-model9.7 | -42.4123 |
|  | XP\_065059637\_11091-model9.8 | -42.0774 |
|  | XP\_065059637\_11091-model9.9 | -41.2487 |
| # cluster 10 - Size: 1 |  |  |
|  | XP\_065059637\_11091-model10 | -46.1889 |
| # cluster 11 - Size: 14 |  |  |
|  | XP\_065059637\_11091-model11 | -46.1282 |
|  | XP\_065059637\_11091-model11.1 | -45.9739 |
|  | XP\_065059637\_11091-model11.2 | -45.471 |
|  | XP\_065059637\_11091-model11.3 | -44.85 |
|  | XP\_065059637\_11091-model11.4 | -44.6129 |
|  | XP\_065059637\_11091-model11.5 | -44.3381 |
|  | XP\_065059637\_11091-model11.6 | -44.2618 |
|  | XP\_065059637\_11091-model11.7 | -44.2318 |
|  | XP\_065059637\_11091-model11.8 | -43.9816 |
|  | XP\_065059637\_11091-model11.9 | -43.6184 |
|  | XP\_065059637\_11091-model11.10 | -43.4981 |
|  | XP\_065059637\_11091-model11.11 | -42.5292 |
|  | XP\_065059637\_11091-model11.12 | -42.232 |
|  | XP\_065059637\_11091-model11.13 | -42.0509 |
| # cluster 12 - Size: 4 |  |  |
|  | XP\_065059637\_11091-model12 | -46.0455 |
|  | XP\_065059637\_11091-model12.1 | -46.0335 |
|  | XP\_065059637\_11091-model12.2 | -43.2946 |
|  | XP\_065059637\_11091-model12.3 | -42.2358 |
| # cluster 13 - Size: 1 |  |  |
|  | XP\_065059637\_11091-model13 | -45.6739 |
| # cluster 14 - Size: 3 |  |  |
|  | XP\_065059637\_11091-model14 | -45.5597 |
|  | XP\_065059637\_11091-model14.1 | -43.8492 |
|  | XP\_065059637\_11091-model14.2 | -43.0294 |
| # cluster 15 - Size: 7 |  |  |
|  | XP\_065059637\_11091-model15 | -45.5122 |
|  | XP\_065059637\_11091-model15.1 | -44.7988 |
|  | XP\_065059637\_11091-model15.2 | -44.793 |
|  | XP\_065059637\_11091-model15.3 | -43.6214 |
|  | XP\_065059637\_11091-model15.4 | -43.5608 |
|  | XP\_065059637\_11091-model15.5 | -43.4422 |
|  | XP\_065059637\_11091-model15.6 | -42.836 |
| # cluster 16 - Size: 2 |  |  |
|  | XP\_065059637\_11091-model16 | -45.363 |
|  | XP\_065059637\_11091-model16.1 | -42.3903 |
| # cluster 17 - Size: 3 |  |  |
|  | XP\_065059637\_11091-model17 | -45.3536 |
|  | XP\_065059637\_11091-model17.1 | -41.8975 |
|  | XP\_065059637\_11091-model17.2 | -39.1536 |
| # cluster 18 - Size: 2 |  |  |
|  | XP\_065059637\_11091-model18 | -44.4335 |
|  | XP\_065059637\_11091-model18.1 | -43.6857 |
| # cluster 19 - Size: 1 |  |  |
|  | XP\_065059637\_11091-model19 | -43.9426 |
| # cluster 20 - Size: 1 |  |  |
|  | XP\_065059637\_11091-model20 | -42.885 |
| # cluster 21 - Size: 1 |  |  |
|  | XP\_065059637\_11091-model21 | -42.8532 |
| # cluster 22 - Size: 1 |  |  |
|  | XP\_065059637\_11091-model22 | -42.7189 |
| # cluster 23 - Size: 3 |  |  |
|  | XP\_065059637\_11091-model23 | -42.4965 |
|  | XP\_065059637\_11091-model23.1 | -42.3286 |
|  | XP\_065059637\_11091-model23.2 | -41.6996 |
| # cluster 24 - Size: 3 |  |  |
|  | XP\_065059637\_11091-model24 | -42.4643 |
|  | XP\_065059637\_11091-model24.1 | -41.825 |
|  | XP\_065059637\_11091-model24.2 | -39.7036 |
| # cluster 25 - Size: 1 |  |  |
|  | XP\_065059637\_11091-model25 | -41.108 |
| # cluster 26 - Size: 1 |  |  |
|  | XP\_065059637\_11091-model26 | -39.6007 |
|  |  |  |
